# Is Carvedilol Effective in Preventing and Modulating Concentric Cardiac Remodelling? A Comprehensive Systematic Review and Meta-Analyses

**DOI:** 10.1155/cdr/9108324

**Published:** 2025-10-28

**Authors:** Alice Valeria Wiyono, Azizah Puspitasari Ardinal

**Affiliations:** Faculty of Life Sciences & Medicine, King's College London, London, UK

**Keywords:** carvedilol, concentric remodelling, diastolic dysfunction, HFpEF

## Abstract

**Background:**

Carvedilol, commonly used to treat hypertension and known for its vasodilatory and pleiotropic effects, has been studied in various patient populations. However, its specific impact on diastolic dysfunction and heart failure with preserved ejection fraction (HFpEF) remains unclear.

**Aim:**

The aim of the study is to evaluate carvedilol's efficacy in preventing concentric cardiac remodelling in at-risk individuals and modulating it in patients with HFpEF.

**Methods:**

In adherence to PRISMA guidelines, we searched PubMed and ScienceDirect up to March 2024 using terms related to carvedilol and HFpEF. We included randomised controlled trials and prospective cohort studies published in English. Outcomes include changes in natriuretic peptides and echocardiography parameters of diastolic function. Exclusion criteria encompassed non-English studies, nonhuman studies and studies not using carvedilol or exclusively involving HFrEF patients. Risk of bias was assessed using the revised Cochrane tool and Newcastle–Ottawa Scale. Data synthesis was performed using a random-effects meta-analysis with sensitivity analyses and a leave-one-out procedure to explore heterogeneity.

**Results:**

Eighteen studies involving 2233 participants were included. Various populations were included: those with HFpEF or undergoing cardiotoxic chemotherapy. Meta-analysis did not reveal significant effects of carvedilol on echocardiography parameters such as E/A ratio (mean difference 0.04, 95% CI −0.01 to 0.08), E/e⁣′ ratio (mean difference −0.50, 95% CI −1.39 to 0.39) and LVMI (mean difference 0.21, 95% CI −3.13 to 3.55), with substantial heterogeneity observed in LVEF, LVMI and BNP.

**Conclusion:**

Carvedilol does not significantly impact diastolic dysfunction across various populations. However, the diversity of study populations and outcomes contributes to the heterogeneity of results.

## 1. Introduction

Heart failure with preserved ejection fraction (HFpEF) presents a range of pathophysiological mechanisms, each demanding specific treatment strategies. These may encompass hypertension, cardiac amyloidosis, valvular heart disease, hypertrophic obstructive cardiomyopathy and coronary artery disease (CAD). Discovering evidence-based therapy for patients suffering from HFpEF has been challenging. Trials including those evaluating angiotensin receptor-neprilysin inhibitors (ARNIs), angiotensin-converting enzyme inhibitors (ACEis), angiotensin-receptor blockers (ARBs), mineralocorticoid receptor antagonists (MRAs) and digoxin have fallen short in demonstrating advantages in their primary endpoints across HFpEF patients [1–6]. Therefore, guidelines for HFpEF primarily address symptom relief and managing associated conditions that could worsen the heart [7, 8].

Several trials demonstrated the significant benefit of *β*B in improving outcomes in heart failure with reduced ejection fraction (HFrEF) patients. Thus, it receives a Class I recommendation and is included in the four-pillar therapy regimen for HFrEF [7, 8]. Conversely, in HFpEF, *β*B receives a Class IIB recommendation specifically for managing hypertension as a comorbid condition. Furthermore, the TOPCAT, CHARM-Preserved, I-PRESERVE and PARAMOUNT trials may offer insights into *β*B utilisation via subgroup analyses, given that certain participants received beta-blockers concurrently [1, 3–5]. However, multicentre randomised clinical trials solely dedicated to *β*B therapy in the HFpEF population, particularly those without CAD and atrial fibrillation, remain scarce.

Numerous meta-analyses have yielded conflicting results regarding the clinical efficacy of *β*B in reducing mortality and heart failure (HF) hospitalisation in the overall HFpEF population. Whilst some meta-analyses have failed to demonstrate significant benefits [9, 10], others suggest a potential reduction in the mortality advantage of *β*B in HFpEF, albeit without a notable effect on reducing hospitalisation rates [11–13]. Moreover, subgroup analysis from the SENIOR trial has demonstrated the comparable benefits of nebivolol in elderly patients with HFpEF and HFrEF, indicating its potential efficacy across various ejection fraction spectra owing to its vasodilatory effects [14]. Although there is limited supporting evidence, *β*B remains commonly prescribed for the management of HFpEF in patients without CAD and atrial fibrillation.

*β*Bs are renowned for their ability to counteract adrenergic stimulation of the heart, resulting in decreased inotropy and chronotropy, as well as possessing antiarrhythmic properties. These attributes contribute to their efficacy in reversing remodelling when combined with other guideline-directed medical therapies (GDMT) in HFrEF patients. Moreover, carvedilol, a noncardiac selective *β*B, has more pronounced vasodilatory properties and exhibits pleiotropic effects that may benefit HFpEF patients [15–18]. Additionally, a recent study conducted in rats unveiled the direct impact of *β*B on extracellular matrix remodelling in HF following myocardial infarction (MI) [19]. Therefore, in this study, we aim to evaluate the role of carvedilol in preventing and targeting concentric cardiac remodelling.

## 2. Method

This systematic review adhered to the guidelines outlined in the Preferred Reporting Items for Systematic Reviews and Meta-Analyses (PRISMA) 2020 statement and checklist [20].

### 2.1. Literature Searching

A systematic search was conducted in PubMed and ScienceDirect in March 2024. The keywords used were “carvedilol AND HFpEF,” “carvedilol AND concentric hypertrophy,” “carvedilol AND diastolic heart failure,” “carvedilol AND diastolic dysfunction.” Only studies published in English were considered for inclusion in this systematic review. Duplicate records were removed using EndNote prior to screening.

### 2.2. Study Selection and Data Collection

Each author autonomously conducted searches and screened articles. Initially, study selection entailed evaluating titles and abstracts, followed by assessing full-text reports to determine eligibility. Any discrepancies in study selection were resolved through discussion. The exclusion criteria were (1) studies not published in English, (2) nonresearch articles, (3) nonhuman studies, (4) not using carvedilol for the intervention, (5) only including patients with HFrEF—defined in the guideline [8] and (6) not reporting relevant outcomes. The inclusion criteria were (1) randomised controlled trial or prospective cohort study, (2) enrolled patients with HFpEF or concentric hypertrophy, or normal LVEF (> 40%) but at risk of developing HFpEF or concentric hypertrophy and (3) presented outcomes (at least one of the following): NT-proBNP or BNP, left ventricular mass (LVM) or LVM index (LVMI), or echocardiography parameters related to diastolic dysfunction [21].

### 2.3. Quality Assessment

Both authors independently conducted quality assessments of the studies, resolving any discrepancies through discussion with all authors. The revised Cochrane Risk of Bias Tool 2 was utilised to assess the quality of included randomised controlled trials [22], whilst the Newcastle–Ottawa Scale (NOS) was employed for observational studies [23]. Subsequently, overall scores were computed and categorised as very low, low, moderate or high quality.

### 2.4. Data Extraction

Data extraction encompassed all authors employing a standardised form to extract outcomes from eligible studies. The extracted data from each study included the author's name and year of publication, study design, sample size, mean age, gender distribution, prevalence of comorbidities, intervention dose, follow-up duration and adjusted effect size for outcomes.

### 2.5. Study Outcomes

The outcomes assessed included changes in the mean of NT-proBNP or BNP levels, LVM or LVMI and echocardiography parameters related to diastolic dysfunction [21].

### 2.6. Statistical Analysis

Meta-analyses were conducted using the “metafor” package in R Software Version 4.3.3. The effect sizes were calculated as mean differences (MDs) between carvedilol and placebo or control groups for each parameter (E/A ratio, E/e⁣′ ratio, LVMI, LVEF and BNP). The variances of each MD were calculated based on standard deviation (SD) and sample sizes. A random-effects (RE) model using the DerSimonian and Laird method [24] with restricted maximum likelihood (REML) was utilised to pool the effect sizes, accounting for potential heterogeneity among studies. For studies that reported multiple treatment groups, such as varying dosages of carvedilol, pooled MD and SD were calculated by weighting according to sample size. Heterogeneity was assessed using the *I*^2^ statistic and Cochrane's *Q* test. An *I*^2^ value greater than 50% or a *p* value less than 0.10 in Cochrane's *Q* test indicated significant heterogeneity [25]. Parameters displaying significant heterogeneity were subsequently subjected to sensitivity analysis and leave-one-out analysis [26]. Forest plots were generated to visually represent each study's effect sizes and 95% confidence intervals (CIs) and the overall pooled estimate.

## 3. Results

### 3.1. Study Selection and Characteristics

**Figure 1:**
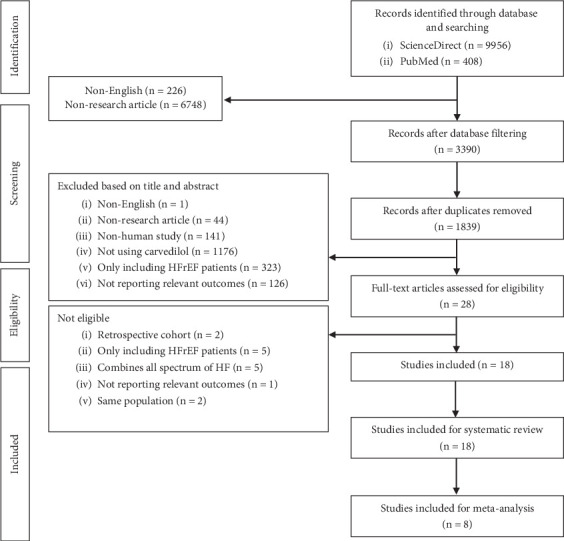
PRISMA flowchart of study selection.

In accordance with the PRISMA 2020 guidelines (Tables S1 and S2), we conducted a systematic review to identify studies examining the effect of carvedilol in preventing and targeting concentric cardiac remodelling. A structured search was carried out across ScienceDirect and PubMed databases, yielding a total of 10,364 records. After adjusting for libraries' filters and removing duplicates, 1839 records were screened. Initial screening was based on title and abstract, excluding numerous articles, resulting in 28 articles being assessed for eligibility. Out of the 28 studies analysed, four exclusively enrolled patients with HFrEF [27–30], one encompassed the entire spectrum of HF [31], two were retrospective cohort studies [32, 33], two shared the same study populations [34, 35] as other studies [30, 36] and one failed to report relevant outcomes [37]. As a result, 18 studies were deemed eligible and included in this systematic review (Figure 1). A total of 2233 participants were included in the study, and their baseline characteristics are summarised in Table 1. This review included a diverse set of populations and conditions, ranging from uncontrolled hypertension, hypertension with left ventricular hypertrophy (LVH), acute myocardial infarction (AMI), cirrhosis and cancer patients undergoing chemotherapy, with a wide age range of participants, spanning from early adulthood to elderly. The gender distribution varied, with some studies focusing exclusively on female populations, such as those with breast cancer [48, 50]. The follow-up periods ranged from 3 months to over 3 years. All studies that satisfied our criteria and reported data in terms of mean with either SD or CI of the pre- and posttreatment or the delta between pre- and posttreatment were included in the meta-analysis.

### 3.2. Quality Assessment

Three prospective cohort studies are included [38, 39, 51], rated from moderate to high quality using NOS. All studies have no study controls for additional factors. There is no nonintervention group in two studies [38, 39]. One study was not followed up long enough for outcomes to occur [38]. The follow-up rate was < 80% in one study (Table S3) [51]. Out of the 15 RCTs that were included, three were found to have some concerns regarding the risk of biases [44, 45, 52], and the remaining RCTs were determined to have a low risk of bias (Table S4). This is mainly attributed to the randomisation process and blinding.

### 3.3. Effect of Carvedilol on Echocardiography Parameters

**Figure 2:**
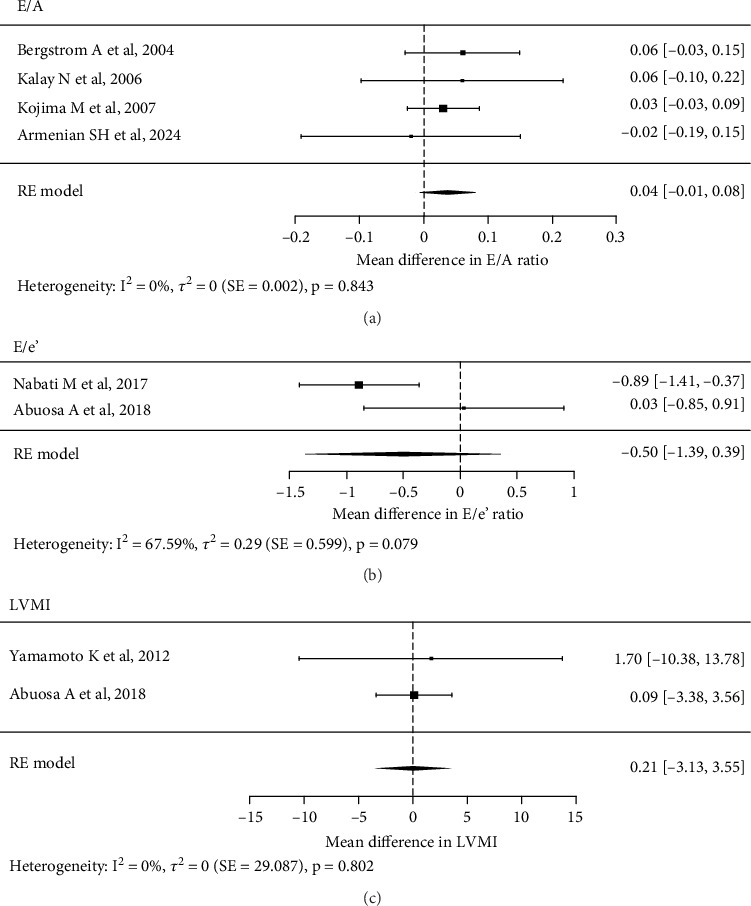
Effect of carvedilol in altering (a) E/A ratio, (b) E/e⁣′ ratio and (c) LVMI.

The pooled analysis of the E/A ratio from four studies with a total of 318 participants [41, 44, 45, 54] found no significant effect of carvedilol compared to control or placebo, with a MD of E/A ratio 0.04 (95% CI −0.01 to 0.08; *p* = 0.105). Heterogeneity was not significant (*I*^2^ = 0%, *p* = 0.843). For the E/e⁣′ ratio, two studies including 245 participants were analysed [48, 49]. The pooled MD E/e ratio change was −0.50 (95% CI −1.39 to 0.39; *p* = 0.270), indicating no significant difference. However, heterogeneity was substantial (*I*^2^ = 67.59%, *p* = 0.0790). The effect of carvedilol on LVMI was evaluated across three studies involving 416 participants [36, 45, 49]. However, a significantly high heterogeneity was observed. Consequently, sensitivity analysis and the leave-one-out approach were conducted, with the study by Kojima et al. [45] being excluded. The pooled RE yielded a value of 0.21 (95% CI −3.13 to 3.55; *p* = 0.901), with no significant heterogeneity detected (*I*^2^ = 0%, *p* = 0.802) (Figure 2). In assessing the effect on LVEF, six studies encompassing 853 participants exhibited very high heterogeneity [36, 45, 48–50, 54]. Following sensitivity analysis and leave-one-out, all studies were excluded, leading to an inconclusive determination regarding the impact of carvedilol on LVEF change in this meta-analysis.

Other echocardiography parameters, such as left ventricular internal diameter (LVID), were reported in only one study; however, the documentation failed to specify whether measurements were taken during systole or diastole [39]. Left ventricular end-diastolic diameter (LVEDD) was reported in two studies [50, 54], though one did not conform to the previously defined reporting standards [50]. Left ventricular diastolic diameter (LVDD) was addressed in two studies, but it lacked clarification on the specific phase of diastole during which measurements were taken [41, 49]. Left ventricular end-systolic diameter (LVESD) was documented in one study [54]. Similarly, left ventricular systolic diameter (LVSD) was reported in one study without specifying the systolic phase [41]. Additionally, interventricular septal thickness (IVST) was also noted in only one study without specifying the cardiac cycle phase [39]. Given these inconsistencies and the absence of detailed measurement context, it is challenging to draw reliable conclusions from these parameters for broader applicative purposes.

### 3.4. Effect of Carvedilol on Natriuretic Peptide

An examination was carried out on the influence of carvedilol on BNP levels, utilising data from six studies involving a collective of 745 participants [36, 41, 42, 45, 50, 54]. Nevertheless, exceedingly substantial heterogeneity was noted. Subsequent sensitivity analysis and application of the leave-one-out method led to the exclusion of all studies. Therefore, we are unable to draw a definitive conclusion regarding the effect of carvedilol on BNP levels in this study.

## 4. Discussion

The diverse populations and conditions included in the study allow a broad evaluation of carvedilol's effects across different cardiac stressors, whether it has a cardioprotective effect in preventing concentric remodelling in patients exposed to substance-promoting remodelling such as anthracycline, reduces remodelling in patients experiencing LVH due to MI or hypertension, or provides no benefit at all. In this study, we observed that carvedilol did not show any advantage over a placebo in changing the E/A ratio. Among the studies analysed, two involved cancer patients treated with anthracycline [44, 54]. The remaining two studies focused on patients with HFpEF: Kojima et al. enrolled patients with LVEF ≥ 50% and an increase in BNP level [45], whilst Bergstrom et al. included patients with LVEF > 45% and clinical signs and symptoms of HF [41]. A pooled analysis of these four studies revealed that carvedilol does not seem to offer any protective effect against diastolic dysfunction, and there is no discernible benefit of carvedilol in mitigating the ongoing or developed diastolic dysfunction. The pooled analysis of two studies on the E/e⁣′ ratio yields similar findings, indicating that carvedilol does not appear to provide any protective effect against diastolic dysfunction, particularly among cancer patients undergoing chemotherapy. The pooled analysis of two studies on LVMI following carvedilol treatment revealed no benefit of carvedilol in altering LVMI, neither in reducing nor preventing remodelling.

Whilst some studies align with our results, they predominantly focus on mortality and composite cardiac outcomes, whereas our investigation centres on concentric remodelling as assessed by echocardiographic parameters. CAPITAL-RCT indicated that extended carvedilol therapy may not confer advantages for individuals with ST-elevation MI (STEMI) and LVEF ≥ 40% who undergo percutaneous coronary intervention (PCI), despite the study's limited statistical power [55]. Moreover, a post hoc analysis of CAPITAL-RCT found that carvedilol significantly decreased composite cardiac outcomes compared to control in patients with heart failure and mildly reduced ejection fraction (HFmrEF), but it did not show any benefit in patients with normal LVEF [56]. A subgroup analysis of the J-DHF study indicates that the composite outcome was not decreased by low-dose carvedilol treatment, whilst a favourable trend was observed in the standard-dose treatment, although it was not statistically significant [36]. A meta-analysis investigated the effect of *β*B treatment in STEMI patients with preserved LVEF following primary PCI. The findings suggest that prolonged oral B-blocker does not reduce mortality but does decrease the incidence of recurrent MI and angina in the short run [57]. A comparison between carvedilol and nebivolol in elderly HFpEF patients, identified by LVEF ≥ 40%, revealed that neither *β*Bs demonstrated improvement in diastolic dysfunction through echocardiographic evaluation [53]. Some HFpEF patients may exhibit diminished responsiveness of *β*B or a blunted response of *β* receptors [58]. Moreover, underdosing medication is prevalent in elderly patients due to intolerance issues [59], whilst the effectiveness of *β*Bs is known to be influenced by dosage, which may be the key determinant in this context [60].

Despite the findings in our meta-analyses, several studies included in this systematic review favour the opposite. The SWEDIC trial demonstrated that carvedilol therapy improves the E/A ratio and E wave velocity compared to placebo in patients with diastolic HF, especially those with a heart rate of ≥ 71 bpm. This implies an improvement in early diastolic filling among these patients despite no change in other parameters such as deceleration time (DT), isovolumetric relaxation time (IVRT) and pulmonary vein systolic to diastolic velocity ratio (pv SD). Additionally, they noted a tendency towards enhanced exercise capacity [41]. Similar findings were reported by Lam et al., which showed that high-dose *β*B significantly lowers mortality risk in patients with HFpEF and elevated heart rate [33]. This was reinforced by findings from Takeda et al., demonstrating a decline in BNP levels and enhanced exercise capacity following 12 months of carvedilol therapy, indicating reduced neurohumoral activity [42]. This could be attributed to the principal mechanisms through which *β*B deliver its therapeutic advantages, involving mitigating the cardiotoxic impacts of catecholamines, thereby decreasing myocardial oxygen demand and enhancing cardiac function. Besides, through heart rate reduction, carvedilol increases diastolic filling time. Additionally, *β*B also diminishes the occurrence of HF over extended observation periods [57, 61]. Domagoj et al. observed that patients with HFpEF defined by LVEF > 40% administered carvedilol had a higher overall survival rate, improvement in New York Heart Association (NYHA) class and LVEF [51]. Despite the improved clinical outcome, these findings may be explained by some participants experiencing an enhancement in LVEF. It is possible that they had previously been diagnosed with HFrEF, a condition now classified as heart failure with improved ejection fraction (HFimpEF) according to the latest guidelines [8].

Animal model studies indicated that *β*B increased the survival of the hypertensive HFpEF rat model [62, 63]. An RCT included in this review also indicates the use of carvedilol for essential hypertension treatment and shows a favourable effect on peak filling rate index (PFRI) during exercise, suggesting an improvement in diastolic function [38]. Studies conducted on hypertensive patients with LVH treated with carvedilol indicate its effectiveness in lowering blood pressure and regressing LVM [39, 43, 64], although renin-angiotensin-aldosterone blockers were superior [64]. The beneficial effect on HFpEF associated with hypertension could stem from the additional alpha-blocking effect of carvedilol, which decreases the afterload, consequently improves cardiac output and reduces the myocardial oxygen demand. Other researchers propose that carvedilol may enhance the clinical outcome of HFpEF patients by preventing or reversing LV dilatation to a certain degree whilst concurrently restoring diastolic function [47]. Interestingly, in addition to showing evidence of LVM regression, Xiaozhen et al. observed the protective effect of carvedilol on coronary endothelial dysfunction in hypertensive patients with LVH. This action involves modulation of endothelin (ET-1) and nitric oxide (NO), subsequently enhancing coronary flow rate even in the absence of underlying CAD [43]. The findings suggest that carvedilol may affect the remodelling of the coronary endothelium. In line with this, an animal model noted the dose-dependent anti-inflammatory effect of *β*B in HFpEF, independent of the haemodynamic effect [62]. As mentioned previously, carvedilol exerts a dose-dependent effect. When administered at dosages that do not lower systemic blood pressure, it has been noted to inhibit cardiac hypertrophy and remodelling in rats with hypertension [65].

Four studies evaluated the protective function of carvedilol in averting diastolic dysfunction among cancer patients without pre-existing HF [44, 48, 50, 54]. Carvedilol, administered at varying doses, has exhibited a protective role in maintaining diastolic function in individuals undergoing cardiotoxic chemotherapy [44, 48, 50]. Carvedilol may exert its cardioprotective effect because of its potent antioxidant property, as evidenced by its ability to decrease the presence of free oxygen radicals in HF [66, 67]. Additionally, cardiotoxic drugs like anthracycline can trigger calcium release in cardiomyocytes [68]. Carvedilol enhances the activity of sarcoplasmic reticulum calcium ATPase and prevents its downregulation, regardless of its beta-blocking effect [69]. Furthermore, carvedilol has been shown to alleviate doxorubicin-induced damage by diminishing oxidative stress and apoptosis [70, 71]. However, one study assessing standardised left ventricular wall thickness–dimension ratio *Z* score, E/A⁣′ ratio, BNP and NT-proBNP did not reveal improvement using low-dose carvedilol. Nevertheless, their results suggested that carvedilol was most effective in individuals who survived for longer durations, indicating potentially more severe cardiotoxicity in this specific subgroup [54].

Whilst we were unable to observe a direct cardiac impact of carvedilol in preventing or reversing remodelling among patients with preserved LVEF or at risk of diastolic dysfunction in our pooled analyses, it does not necessarily imply that carvedilol is ineffective in HFpEF or in preventing diastolic dysfunction. Carvedilol may exert its influence indirectly by reducing myocardial demand through its beta-blocking activity and lowering afterload. This study might show negative results due to the heterogeneity of the interstudy population in the pooled analyses. It is also worth mentioning that the direct protective effect of carvedilol in HFpEF remains uncertain, given that certain studies mentioned earlier demonstrated favourable outcomes associated with anti-inflammatory properties.

### 4.1. Study Limitations and Suggestions for Further Research

Each meta-analysis in this study includes only a small number of studies. Additionally, we did not differentiate between patients at risk of concentric remodelling but without underlying heart disease, such as cancer patients undergoing chemotherapy, and patients who already experienced HFpEF. Subgroup analysis was not feasible due to the limited number of studies included, each with diverse echocardiographic parameters as outcomes. Therefore, further studies could focus on addressing the specific subgroups mentioned, such as differentiating between patients at risk of concentric remodelling and patients already experiencing HFpEF. Further investigation related to dose-dependent outcomes is also necessary. Increasing the number of studies within each subgroup could allow for more robust subgroup analyses. Moreover, although the latest guideline has segmented HF into categories such as HFrEF, HFmrEF, HFpEF and HFimpEF, defining HFpEF as LVEF ≥ 50% [8], our study continues to utilise the LVEF cutoff of > 40% because of the scarcity of studies since the latest guideline was formulated within the past 3 years. Therefore, there is a need for more RCTs aligning with this criterion.

## 5. Conclusion

This systematic review and meta-analysis assessed the effect of carvedilol on reversing and preventing concentric cardiac remodelling or diastolic dysfunction. Despite the indirect benefit in LVH due to hypertension through its beta- and alpha-blocking abilities and its known radical-scavenging properties, our findings indicate that carvedilol does not significantly influence echocardiographic parameters such as E/A ratio, E/e⁣′ ratio and LVMI. This suggests that there is no substantial benefit of carvedilol on diastolic dysfunction across studied populations. However, given the diversity of the study populations—which included elderly patients with LVH and hypertension, as well as cancer patients undergoing cardiotoxic chemotherapy without pre-existing heart disease, our findings should be interpreted cautiously, and future studies should focus on more homogenous patient groups or perform subgroup analyses to better understand the conditions under which carvedilol might be effective.

## Figures and Tables

**Table 1:** Baseline characteristics of the included studies.

**Author, year**	**Design**	**Sample size**	**Population**	**HFpEF or hypertrophy definition**	**Mean age (min–max)**	**HT (%)**	**DM (%)**	**IHD (%)**	**AFib/Aflut (%)**	**Sex, female (%)**	**Intervention**	**Follow-up duration**	**Relevant outcomes**
Herber et al., 1987 [38]	Prospective cohort	12	Uncontrolled hypertensive	Unclear	53 (45–64)	12 (100.0)	N/A	N/A	N/A	5 (41.7)	Carvedilol 25 mg BID	4 weeks	PFRI, LVEF
Verza et al., 1996 [39]	Prospective cohort	22	Hypertensive elderly + LVH (increased LVMI)	LVMI ≥ 130 g/m^2^ for men and ≥ 110 g/m^2^ for women	69 (61–77)	N/A	N/A	N/A	N/A	N/A	Carvedilol 25 mg QD	6 months	LVMI, PWT, IVST, LVID
Basu et al., 1997 [40]	RCT	146	AMI	Unclear	60 (53–70)	N/A	N/A	33 (22.6)	N/A	23 (15.8)	Carvedilol 12.5 or 25 mg BID vs. placebo	168 days	E/A, LVEF
Bergstrom et al., 2004 [41]	RCT	97	General	Signs and/or symptoms of HF, LVEF ≥ 45%, evidence of abnormal diastolic function	66 (48–84)	64 (66.0)	14 (14.4)	11 (11.3)	N/A	42 (43.3)	Carvedilol 25 or 50 mg BID vs. placebo	6 months	E/A, IVRT, DT, pv S/D, LA, LVDD, LVSD, BNP
Takeda et al., 2004 [42]	RCT	40	General	Framingham criteria, LVEF ≥ 45%	71 (68–74)	24 (60.0)	N/A	21 (53.0)	12 (30.0)	19 (48.0)	Carvedilol 5–10 mg QD vs. placebo	12 months	BNP
Galzerano et al., 2005 [43]	RCT	70	Hypertensive	Unclear	69 (50–70)	60 (100.0)	N/A	N/A	N/A	23 (32.9)	Carvedilol 25 mg QD vs. telmisartan 80 mg QD	44 weeks	LVMI
Kalay et al., 2006 [44]	RCT	50	Malignancy + prospective anthracycline therapy	No HF	48 (33–61)	N/A	N/A	0 (0)	N/A	43 (86)	Carvedilol 12.5 mg QD vs. placebo	6 months	E/A, IVRT, LVEF
Kojima et al., 2007 [45]	RCT	20	Chronic haemodialysis	LVEF > 50% and BNP > 390 ng/L after haemodialysis	63 (59–66)	N/A	N/A	N/A	N/A	11 (55.0)	Carvedilol 10 mg QD vs. placebo	3 months	E/A, LVEF, LVMI, BNP
Miller et al., 2010 [46]	RCT	287	Hypertensive + LVH	LVMI ≥ 134 g/m^2^ for men and ≥ 110 g/m^2^ for women	57 (46–67)	287 (100.0)	58 (20.2)	N/A	N/A	159 (55.4)	Carvedilol/lisinopril 20 or 40 or 80/20 mg QD vs. atenolol/lisinopril 50 or 75 or 100/20 mg QD vs. lisinopril 10 or 20 or 40 mg QD	12 months	LVMI
Xiaozhen et al., 2010 [47]	RCT	57	Hypertensive + LVH	LVMI ≥ 134 g/m^2^ for men and ≥ 110 g/m^2^ for women	61 (48–76)	57 (100.0)	0 (0.0)	57 (100.0)	N/A	22 (38.6)	Carvedilol 10 mg BID vs. metoprolol 50 mg BID	6 months	LVMI
Yamamoto et al., 2013 [36]	RCT	245	General	Framingham criteria, LVEF > 40%	72 (60–83)	197 (90.4)	75 (30.6)	N/A	83 (33.9)	103 (42.0)	Carvedilol 20 mg QD vs. placebo	3.2 years	LVEF, LVMI, BNP
Nabati et al., 2017 [48]	RCT	91	Women with breast cancer underwent anthracycline therapy	No HF	47 (35–59)	16 (17.6)	8 (8.8)	0 (0)	N/A	91 (100.0)	Carvedilol 6.125 mg BID vs. not given	6 months	E/A, E/e⁣′, LA, pv S/D, LVEF
Abuosa et al., 2018 [49]	RCT	154	Cancer patients treated with doxorubicin	No HF	43 (23–60)	18 (11.7)	27 (17.5)	N/A	N/A	112 (72.7)	Carvedilol 6.25 mg QD vs. 12.5 mg QD vs 25 mg QD vs. placebo	6 months	E/A, E/e⁣′, DT, LVDD, LVEF
Avila et al., 2018 [50]	RCT	192	Breast cancer treated with anthracycline, cyclophosphamide or taxane	No HF	52 (41–62)	12 (6.3)	9 (4.7)	N/A	N/A	192 (100.0)	Carvedilol 25 mg BID vs. placebo	24 weeks	Diastolic dysfunction parameters (unspecified), LVEDD, LVEF, BNP
Domagoj et al., 2019 [51]	Prospective cohort	317	General	Signs and/or symptoms of HF, LVEF ≥ 40%, radiographic evidence	75 (66–84)	N/A	N/A	N/A	N/A	163 (51.4)	Carvedilol vs. not given	12 months	LVEF
Premkumar et al., 2020 [52]	RCT	189	Cirrhosis	Echo parameters of diastolic dysfunction defined by AHA	52 (41–61)	29 (15.3)	40 (21.2)	N/A	N/A	N/A	Carvedilol vs. carvedilol + ivabradine vs. control	12 months	E/A, E/e⁣′, BNP
Park et al., 2023 [53]	RCT	62	Elderly	Signs and/or symptoms of HF, LVEF ≥ 40%, NT-proBNP > 300 pg/mL	78 (73–84)	33 (53.2)	17 (27.4)	N/A	0 (0)	25 (40.3)	Carvedilol vs. nebivolol	12 months	E/A, E/e⁣′, LVMI, IVRT, DT, LV GLS, NT-proBNP
Armenian et al., 2024 [54]	RCT	182	Childhood cancer exposed to anthracycline	No HF	24 (20–37)	8 (4.4)	8 (4.4)	N/A	N/A	91 (50.0)	Carvedilol 6.25 mg BID vs. placebo	24 months	E/A, *Z* score, LVMI, LVEDD, LVESD, LVEF, BNP, NT-proBNP

Abbreviations: AFib/Aflut, atrial fibrillation/atrial flutter; AHA, American Heart Association; AMI, acute myocardial infarction; BID, twice a day; BNP, B-type natriuretic peptide; DM, diabetes mellitus; DT, deceleration time; E/A, early to atrial wave velocity ratio; E/e⁣′, early mitral inflow to early mitral annular diastolic velocity ratio; HF, heart failure; HFpEF, heart failure with preserved ejection fraction; HT, hypertension; IHD, ischaemic heart disease; IVRT, isovolumetric relaxation time; IVST, interventricular septal thickness; LA, left atrial size; LVDD, left ventricular diastolic diameter; LVEDD, left ventricular end-diastolic diameter; LVEF, left ventricular ejection fraction; LV GLS, left ventricular global longitudinal strain; LVH, left ventricular hypertrophy; LVID, left ventricular internal diameter; LVMI, left ventricular mass index; LVSD, left ventricular systolic diameter; PFRI, peak filling rate index; pv SD, pulmonary vein systolic to diastolic velocity ratio; PWT, posterior wall thickness; QD, once a day; RCT, randomised controlled trial.

## Data Availability

All data analysed in this review were extracted from previously published studies cited in the article. Any additional materials are available from the corresponding author upon reasonable request.

## Supporting Information

Supporting Information Additional supporting information can be found online in the Supporting Information section. In addition to the main text, we have included supporting information to enhance the clarification of adherence to PRISMA 2020 guidelines and the evaluation of study biases. Table S1 provides a detailed checklist used in accordance with PRISMA 2020 guidelines, ensuring comprehensive reporting of this systematic review and meta-analyses. It lists each required item and its location within our manuscript, helping readers verify our adherence to these guidelines. Table S2 presents the abstract checklist, verifying that our manuscript's abstract complies with the PRISMA guidelines for abstracts, ensuring that all essential components of systematic review reporting are succinctly covered. Table S3 presents the quality assessment of included cohort studies using the Newcastle–Ottawa Scale, which assesses the selection, comparability and outcome of the cohorts studied. Lastly, Table S4 details the risk of bias in included studies as evaluated by the Cochrane Risk of Bias Tool 2, reinforcing the credibility of our review findings.
