# Supplementary material for: Is Carvedilol Effective in Preventing and Modulating Concentric Cardiac Remodelling? A Comprehensive Systematic Review and Meta-Analyses
Source: Cardiovasc Ther. 2025 Oct 28;2025:9108324. doi: 10.1155/cdr/9108324 (PMC12585786; doi:10.1155/cdr/9108324)
Supplement: Supporting Information — Additional supporting information can be found online in the Supporting Information section. In addition to the main text, we have included supporting information to enhance the clarification of adherence to PRISMA 2020 guidelines and the evaluation of study biases. Table S1 provides a detailed checklist used in accordance with PRISMA 2020 guidelines, ensuring comprehensive reporting of this systematic review and meta-analyses. It lists each required item and its location within our manuscript, helping readers verify our adherence to these guidelines. Table S2 presents the abstract checklist, verifying that our manuscript's abstract complies with the PRISMA guidelines for abstracts, ensuring that all essential components of systematic review reporting are succinctly covered. Table S3 presents the quality assessment of included cohort studies using the Newcastle–Ottawa Scale, which assesses the selection, comparability and outcome of the cohorts studied. Lastly, Table S4 details the risk of bias in included studies as evaluated by the Cochrane Risk of Bias Tool 2, reinforcing the credibility of our review findings. [file 9108324.f1.zip › Author response (2).docx]

Dear Editorial Office,

Thank you for the screening notes. We have addressed each point as follows:

1. Author emails

The email addresses have been updated in the submission system:

• Alice Valeria Wiyono — alice.valeriawiyono@gmail.com

• Azizah Puspitasari Ardinal — azizahardinal40@gmail.com

1. Data Availability statement

Updated to reflect the presence of data-based figures. The statement now reads:

All data analysed in this review were extracted from previously published studies cited in the article. Additional materials are available from the corresponding author upon reasonable request.

2. Funding statement mismatch (system vs manuscript)

Please proceed with the manuscript version. We have ensured the submission system now matches the manuscript wording.

3. Affiliation / ROR alignment

- The work was conducted while I was a student at King’s College London (KCL); the affiliation has been updated accordingly and aligned with ROR: https://ror.org/0220mzb33.
- My current institution is listed only as a Present address: Padjajaran University, ROR: https://ror.org/ 00xqf8t64.
- Figures and tables have been revised to meet Wiley standards: using correct file formats and resolutions, with graphs provided as editable files.
