# Supplementary material for: Is Carvedilol Effective in Preventing and Modulating Concentric Cardiac Remodelling? A Comprehensive Systematic Review and Meta-Analyses
Source: Cardiovasc Ther. 2025 Oct 28;2025:9108324. doi: 10.1155/cdr/9108324 (PMC12585786; doi:10.1155/cdr/9108324)
Supplement: Supporting Information — Additional supporting information can be found online in the Supporting Information section. In addition to the main text, we have included supporting information to enhance the clarification of adherence to PRISMA 2020 guidelines and the evaluation of study biases. Table S1 provides a detailed checklist used in accordance with PRISMA 2020 guidelines, ensuring comprehensive reporting of this systematic review and meta-analyses. It lists each required item and its location within our manuscript, helping readers verify our adherence to these guidelines. Table S2 presents the abstract checklist, verifying that our manuscript's abstract complies with the PRISMA guidelines for abstracts, ensuring that all essential components of systematic review reporting are succinctly covered. Table S3 presents the quality assessment of included cohort studies using the Newcastle–Ottawa Scale, which assesses the selection, comparability and outcome of the cohorts studied. Lastly, Table S4 details the risk of bias in included studies as evaluated by the Cochrane Risk of Bias Tool 2, reinforcing the credibility of our review findings. [file 9108324.f1.zip › Supplementary materials.docx]

**SUPPLEMENTARY MATERIALS**

**Supplementary table 1.** PRISMA 2020 checklist

| **Section and topic** | **Item #** | **Checklist item** | **Location where item is reported** |
| --- | --- | --- | --- |
| **Title** | | | |
| Title | 1 | Identify the report as a systematic review. | Title |
| **Abstract** | | | |
| Abstract | 2 | See the PRISMA 2020 for Abstracts checklist (Suppl. Table 2). | See Suppl. Table 2 |
| **Introduction** | | | |
| Rationale | 3 | Describe the rationale for the review in the context of existing knowledge. | Introduction: Paragraphs 2 to 4 |
| Objectives | 4 | Provide an explicit statement of the objective(s) or question(s) the review addresses. | Introduction: Paragraph 4 |
| **Methods** | | | |
| Eligibility criteria | 5 | Specify the inclusion and exclusion criteria for the review and how studies were grouped for the syntheses. | Method: Study selection and data collection |
| Information sources | 6 | Specify all databases, registers, websites, organisations, reference lists and other sources searched or consulted to identify studies. Specify the date when each source was last searched or consulted. | Method: Literature searching |
| Search strategy | 7 | Present the full search strategies for all databases, registers and websites, including any filters and limits used. | Method: Literature searching |
| Selection process | 8 | Specify the methods used to decide whether a study met the inclusion criteria of the review, including how many reviewers screened each record and each report retrieved, whether they worked independently, and if applicable, details of automation tools used in the process. | Method: Study selection and data collection |
| Data collection process | 9 | Specify the methods used to collect data from reports, including how many reviewers collected data from each report, whether they worked independently, any processes for obtaining or confirming data from study investigators, and if applicable, details of automation tools used in the process. | Method: Literature searching, study selection and data collection, data extraction |
| Data items | 10a | List and define all outcomes for which data were sought. Specify whether all results that were compatible with each outcome domain in each study were sought (e.g. for all measures, time points, analyses), and if not, the methods used to decide which results to collect. | Method: Study outcomes, statistical analysis |
|  | 10b | List and define all other variables for which data were sought (e.g. participant and intervention characteristics, funding sources). Describe any assumptions made about any missing or unclear information. | Method: Study selection and data collection |
| Study risk of bias assessment | 11 | Specify the methods used to assess risk of bias in the included studies, including details of the tool(s) used, how many reviewers assessed each study and whether they worked independently, and if applicable, details of automation tools used in the process. | Method: Quality assessment |
| Effect measures | 12 | Specify for each outcome the effect measure(s) (e.g. risk ratio, mean difference) used in the synthesis or presentation of results. | Method: Statistical analysis |
| Synthesis methods | 13a | Describe the processes used to decide which studies were eligible for each synthesis (e.g. tabulating the study intervention characteristics and comparing against the planned groups for each synthesis (item #5)) | Results: Study selection and characteristics |
|  | 13b | Describe any methods required to prepare the data for presentation or synthesis, such as handling of missing summary statistics, or data conversions. | Method: Statistical analysis |
|  | 13c | Describe any methods used to tabulate or visually display results of individual studies and syntheses. | Method: Statistical analysis |
|  | 13d | Describe any methods used to synthesise results and provide a rationale for the choice(s). If meta-analysis was performed, describe the model(s), method(s) to identify the presence and extent of statistical heterogeneity, and software package(s) used. | Method: Statistical analysis |
|  | 13e | Describe any methods used to explore possible causes of heterogeneity among study results (e.g. subgroup analysis, meta-regression). | Method: Statistical analysis |
|  | 13f | Describe any sensitivity analyses conducted to assess robustness of the synthesised results. | Method: Statistical analysis  Results: Effect of carvedilol on echocardiography parameters, effect of carvedilol on natriuretic peptide |
| Reporting bias assessment | 14 | Describe any methods used to assess risk of bias due to missing results in a synthesis (arising from reporting biases). | Method: Quality assessment |
| Certainty assessment | 15 | Describe any methods used to assess certainty (or confidence) in the body of evidence for an outcome. | Method: Statistical analysis |
| **Results** | | | |
| Study selection | 16a | Describe the results of the search and selection process, from the number of records identified in the search to the number of studies included in the review, ideally using a flow diagram. | Results: Study selection and characteristics, figure 1 |
|  | 16b | Cite studies that might appear to meet the inclusion criteria, but which were excluded, and explain why they were excluded. | Results: Study selection and characteristics |
| Study characteristics | 17 | Cite each included study and present its characteristics. | Results: Table 1 |
| Risk of bias in studies | 18 | Present assessments of risk of bias for each included study. | Supplementary materials: Suppl. Table 3 and 4 |
| Results of individual studies | 19 | For all outcomes, present, for each study: (a) summary statistics for each group (where appropriate) and (b) an effect estimate and its precision (e.g. confidence/credible interval), ideally using structured tables or plots. | Results: Figure 2 |
| Results of syntheses | 20a | For each synthesis, briefly summarise the characteristics and risk of bias among contributing studies. | Results: Table 1 |
|  | 20b | Present results of all statistical syntheses conducted. If meta-analysis was done, present for each the summary estimate and its precision (e.g. confidence/credible interval) and measures of statistical heterogeneity. If comparing groups, describe the direction of the effect. | Results: Figure 2 |
|  | 20c | Present results of all investigations of possible causes of heterogeneity among study results. | Results: Effect of carvedilol on echocardiography parameters, effect of carvedilol on natriuretic peptide  Discussion: Paragraph 6, study limitations and suggestions for further research |
|  | 20d | Present results of all sensitivity analyses conducted to assess the robustness of the synthesised results. | Results: Effect of carvedilol on echocardiography parameters, effect of carvedilol on natriuretic peptide |
| Reporting biases | 21 | Present assessments of risk of bias due to missing results (arising from reporting biases) for each synthesis assessed. | Results: Quality assessment |
| Certainty of evidence | 22 | Present assessments of certainty (or confidence) in the body of evidence for each outcome assessed. | Results: Figure 2 |
| **Discussion** | | | |
| Discussion | 23a | Provide a general interpretation of the results in the context of other evidence. | Discussion: Paragraphs 1 to 6 |
|  | 23b | Discuss any limitations of the evidence included in the review. | Discussion: Study limitations and suggestions for further research |
|  | 23c | Discuss any limitations of the review processes used. | Discussion: Study limitations and suggestions for further research |
|  | 23d | Discuss implications of the results for practice, policy, and future research. | Discussion: Paragraph 6, study limitations and suggestions for further research |
| **Other information** | | | |
| Registration and protocol | 24a | Provide registration information for the review, including register name and registration number, or state that the review was not registered. | N/A |
|  | 24b | Indicate where the review protocol can be accessed, or state that a protocol was not prepared. | N/A |
|  | 24c | Describe and explain any amendments to information provided at registration or in the protocol. | N/A |
| Support | 25 | Describe sources of financial or non-financial support for the review, and the role of the funders or sponsors in the review. | Acknowledgement |
| Competing interests | 26 | Declare any competing interests of review authors. | Conflict of interest |
| Availability of data, code, and other materials | 27 | Report which of the following are publicly available and where they can be found: template data collection forms; data extracted from included studies; data used for all analyses; analytic code; any other materials used in the review. | References |

**Supplementary table 2.** PRISMA 2020 for Abstracts checklist

| **Section and topic** | **Item #** | **Checklist item** | **Yes/ No** |
| --- | --- | --- | --- |
| **Title** | | | |
| Title | 1 | Identify the report as a systematic review. | Yes |
| **Background** | | | |
| Objectives | 2 | Provide an explicit statement of the main objective(s) or question(s) the review addresses. | Yes |
| **Methods** | | | |
| Eligibility criteria | 3 | Specify the inclusion and exclusion criteria for the review. | Yes |
| Information sources | 4 | Specify the information sources (e.g. databases, registers) used to identify studies and the date when each was last searched. | Yes |
| Risk of bias | 5 | Specify the methods used to assess risk of bias in the included studies. | Yes |
| Synthesis of results | 6 | Specify the methods used to present and synthesise results. | Yes |
| **Results** | | | |
| Included studies | 7 | Give the total number of included studies and participants and summarise relevant characteristics of studies. | Yes |
| Synthesis of results | 8 | Present results for main outcomes, preferably indicating the number of included studies and participants for each. If meta-analysis was done, report the summary estimate and confidence/credible interval. If comparing groups, indicate the direction of the effect (i.e. which group is favoured). | Yes |
| **Discussion** | | | |
| Limitations of evidence | 9 | Provide a brief summary of the limitations of the evidence included in the review (e.g. study risk of bias, inconsistency and imprecision). | Yes |
| Interpretation | 10 | Provide a general interpretation of the results and important implications. | Yes |
| **Other** | | | |
| Funding | 11 | Specify the primary source of funding for the review. | No |
| Registration | 12 | Provide the register name and registration number. | N/A |

**Supplementary table 3.** Newcastle-Ottawa Quality Assessment Scale for cohort studies

| **Assessment** | Herber ME et al | Verza M et al | Domagoj M et al |
| --- | --- | --- | --- |
| **Selection** | | | |
| Representativeness of the intervention cohort | | | |
| a. Truly representative of hypertensive with HFpEF patients* | √ | √ | √ |
| b. Somewhat representative of hypertensive with HFpEF patients* |  |  |  |
| c. Selected group of participants |  |  |  |
| d. No description of the derivation of cohort |  |  |  |
| Selection of the non-intervention cohort | | | |
| a. Drawn from the same community as the intervention cohort* |  |  | √ |
| b. Drawn from a different source |  |  |  |
| c. No description of the derivation of the non-intervention cohort | √ | √ |  |
| Ascertainment of intervention | | | |
| a. Secure record* | √ | √ | √ |
| b. Structured interview* |  |  |  |
| c. Written self-report |  |  |  |
| d. No description |  |  |  |
| Demonstration that outcome of interest was not present at the start of the study | | | |
| a. Yes* | √ | √ | √ |
| b. No |  |  |  |
| **Comparability** | | | |
| Comparability of cohorts on the basis of the design or analysis | | |  |
| a. Study controls for age, medications, gender, comorbidities* | √ | √ | √ |
| b. Study controls for any additional factors (e.g. ethnicity, baseline functional status, socio-economic status)* |  |  |  |
| **Outcome** | | | |
| Assessment of outcome | | | |
| a. Independent blind assessment* |  |  | √ |
| b. Record linkage* | √ | √ |  |
| c. Self-report |  |  |  |
| d. No description |  |  |  |
| Was follow-up long enough for outcomes to occur | | |  |
| a. Yes (≥6 months)* |  | √ | √ |
| b. No (<6 months) | √ |  |  |
| Adequacy of follow-up of cohorts | | |  |
| a. Complete follow-up of all subjects* | √ | √ |  |
| b. Subjects lost to follow-up unlikely to introduce bias: number lost ≤20%, or description provided of the lost* |  |  |  |
| c. Follow-up rate <80%, and no description of those lost |  |  | √ |
| d. No statement |  |  |  |
| **Quality** | **Moderate** | **High** | **High** |

*For each component checked, a score of 1 is assigned

Abbreviations: HFpEF, Heart failure with preserved ejection fraction

**Supplementary table 4.** Cochrane risk of bias

| **Study** | **1** | **2** | **3** | **4** | **5** | **Overall** |
| --- | --- | --- | --- | --- | --- | --- |
| Basu S et al | L | L | L | L | L | Low |
| Bergstrom A et al | L | L | L | L | L | Low |
| Takeda Y et al | L | L | L | L | L | Low |
| Galzerano D et al | L | L | L | L | L | Low |
| Kalay N et al | S | L | L | L | L | Some concerns |
| Kojima M et al | S | L | L | S | L | Some concerns |
| Miller A et al | L | L | L | L | L | Low |
| Xiaozhen H et al | L | L | L | L | L | Low |
| Yamamoto K et al | L | L | L | L | L | Low |
| Nabati M et al | L | L | L | L | L | Low |
| Abuosa A et al | L | L | L | L | L | Low |
| Avila MS et al | L | L | L | L | L | Low |
| Premkumar M et al | S | L | L | L | L | Some concerns |
| Park K et al | L | L | L | L | L | Low |
| Armenian SH et al | L | L | L | L | L | Low |

1 = Bias arising from randomisation process, 2 = Bias due to deviations from intended interventions, 3 = Bias due to missing outcome data, 4 = Bias in measurement of the outcome, 5 = Bias in selection of the reported result. Abbreviations: L, low; H, high; S, some concerns
